# Integrated Neurosurgical Management of Retroperitoneal Benign Nerve Sheath Tumors

**DOI:** 10.3390/cancers15123138

**Published:** 2023-06-10

**Authors:** Alberto Benato, Quintino Giorgio D’Alessandris, Marino Murazio, Fabio Pacelli, Pier Paolo Mattogno, Eduardo Fernández, Liverana Lauretti

**Affiliations:** 1 Rome Campus, Università Cattolica del Sacro Cuore, 00168 Rome, Italy; 2 Department of Neurosurgery, Fondazione Policlinico Universitario Agostino Gemelli IRCCS, 00168 Rome, Italy; 3 Department of Surgery, Fondazione Policlinico Universitario Agostino Gemelli IRCCS, 00168 Rome, Italy

**Keywords:** nerve sheath tumor, neurosurgery, retroperitoneal, peripheral nerve surgery, exoscope

## Abstract

**Simple Summary:**

Surgery for retroperitoneal benign nerve sheath tumors (PNST) is often conducted without microsurgical techniques, as evident from the literature. However, this is associated with a risk of permanent postoperative neurological deficits when lesions arise from relevant retroperitoneal nerve trunks. Among the papers describing micro/neurosurgical approaches to such tumors, only a few report more than five patients. Here, we present our interdisciplinary approach to retroperitoneal PNST in a series of 15 benign retroperitoneal tumors arising from major lumbosacral nerve trunks. The combination of expertise from both neurosurgeons and abdominal surgeons led to good neurological and oncological outcomes. Our data provide further confirmation that such an interdisciplinary approach is the optimal strategy to manage benign retroperitoneal nerve sheet tumors.

**Abstract:**

Peripheral nerve sheath tumors (PNST) of the retroperitoneum are rare and are often treated by general surgeons dealing with retroperitoneal cancers. However, resection without the correct microsurgical technique can cause permanent neurological deficits and pain. Here, we discuss our interdisciplinary approach based on the integration of expertise from neurosurgery and abdominal surgery, allowing for both safe exposure and nerve-sparing microsurgical resection of these lesions. We present a series of 15 patients who underwent resection of benign retroperitoneal or pelvic PNST at our institution. The mean age of patients was 48.4 years; 67% were female. Tumors were 14 schwannomas and 1 neurofibroma. Eight patients (53%) reported neurologic symptoms preoperatively. The rate of complete resection was 87% (n = 13); all symptomatic patients showed improvement of their preoperative symptoms. There were no postoperative motor deficits; one patient (7%) developed a permanent sensory deficit. At a mean postoperative follow-up of 31 months, we observed no recurrences. To our best knowledge, this is the second-largest series of benign retroperitoneal PNST consistently managed with microsurgical techniques. Our experience confirms that interdisciplinary management allows for safe treatment of these tumors with good neurological and oncological outcomes.

## 1. Introduction

The neurosurgical approach to benign nerve sheath tumors (PNST) is based on the application of specific microsurgical techniques that respect healthy nerve fascicles and aim at preserving neurological function [1]. However, while most PNST can be accessed with approaches that are part of the standard armamentarium of peripheral nerve surgeons, the management of tumors occurring in unfamiliar anatomical locations can be associated with a significant increase in surgical complexity. A challenging example is the PNST of the retroperitoneal and pelvic spaces, which represent less than 3–6% of all PNST [2,3,4]. As evident from the literature, these tumors are often treated by surgeons who are familiar with the abdominal district but have limited experience in nerve-sparing microsurgical techniques. Therefore, in many publications, the focus is put on the radicality of resection rather than the preservation of neurological function [5,6,7] and neurological outcomes are under-reported [5,8].

Damage to parent nerves can cause postoperative deficits and severe pain, with a significant impact on patients’ quality of life. The key to improving neurological outcomes is to adopt a multidisciplinary approach that allows for the integration of complementary surgical perspectives, gathering expertise from both peripheral nerve surgery and retroperitoneal surgery.

In the past years, this philosophy has been followed at our institution in the treatment of 15 patients affected by retroperitoneal and pelvic PNST. Herein, we share our experience in the management of these cases and review the pertinent literature.

## 2. Materials and Methods

We retrospectively reviewed the charts of patients treated for retroperitoneal lumbo–-sacral PNST at our institution. The study was approved by the Institutional Ethical Committee (protocol N° 3787); all patients provided written informed consent for the anonymized publication of their data. For each patient, we noted demographics, comorbidities, tumor histology, location and size, preoperative symptoms, surgical approach, surgical time, intraoperative blood loss, complications related to surgery, neurological outcome, and follow-up.

In all surgeries, the microsurgical part was performed by the authors with the greatest experience in peripheral nerve surgery (L.L., E.F.), while the approach to the lesion and the closure of the abdomen were performed by a general surgeon or a gynecologist. All surgeries were conducted under neurophysiological nerve monitoring with EMG of the lower extremities; the use of muscle paralyzing agents was restricted to the surgical steps performed by general surgeons.

Additionally, we performed a systematic review of the MEDLINE database, using the following search string: (“schwannoma” OR “neurofibroma” OR “nerve sheath” OR “neurilemmoma”) AND (“retroperitoneal” OR “pelvic”).

## 3. Results

### 3.1. Original Surgical Series

The mean age of our 15 patients was 48.4 years (range 17–75). The gender distribution was 67% female and 33% male. Histology was schwannoma in 14 cases (93%) and neurofibroma in 1 case (7%). The mean tumor size was 58 mm (range 20–130). All lesions were sporadic; there were no NF1 or NF2 patients in our series.

The diagnosis was incidental in four patients (27%). Four patients (27%) reported abdominal pain, with one of them (7%) also presenting with a palpable mass; seven patients (47%) reported pain and/or numbness in the territory of the affected nerve or a nearby nerve trunk. One patient (7%) reported sciatic pain due to sciatic nerve compression by a femoral nerve schwannoma. In asymptomatic cases, indications for surgery were: (1) documented growth during initial follow-up; or (2) the patient’s preference.

Preoperative biopsies have been obtained in five patients: in three cases, an ultrasound-guided needle biopsy was performed due to diagnostic uncertainties; in two cases, an incisional biopsy of the lesions had been performed during previous gynecologic surgeries that led to the incidental discovery of the PNST (salpingo-oophorectomy, removal of a borderline ovarian tumor). In all biopsied cases, histology confirmed schwannoma after tumor removal. Postoperatively, patients were followed for a mean of 31 months (range 1–108).

The surgical approach and tumor exposure were performed by a general surgeon or a gynecologist. When the lesion was situated medially to the psoas muscle (n = 10), we adopted a median transperitoneal route via a midline abdominal incision, whose size was determined according to the relationship of the tumor to the sacral promontory. For lesions located at or above the sacral promontory (n = 3), the umbilicus was at the lower third of the incision. For lesions located below, the umbilicus was at the upper third (n = 6), but in one case we reopened a preexisting Pfannenstiel incision performed during previous pelvic surgery (see above). When lesions were located laterally to the psoas (n = 5), we followed a lateral retroperitoneal route via an oblique lateral abdominal incision.

In 13 cases, the microsurgical step was performed with the aid of an operating microscope (Leica Microsystems, Wetzlar, Germany). In two cases, we used an exoscope (Aesculap AEOS, B. Braun Melsungen AG, Melsungen, Germany).

The mean surgical time was 300 min. Complete resection was obtained in 13 (87%) cases. In one case, the patient suffered significant blood loss due to continuous oozing during the removal of a giant tumor that hampered the complete removal; surgery was interrupted, and a remnant was left. In another case, due to abundant adhesions generated by previous gynecologic surgery, complete tumor resection could not be achieved without risking nerve injury, and thus a small residue was left.

Excluding the case described above, in which bleeding exceeded 3000 mL, the mean blood loss was 350 mL (Table 1); when including the outlier, the mean blood loss was 540 mL. No other intraoperative complications were encountered.

Postoperatively, no motor deficits in the lower limbs were observed. All patients who experienced pain preoperatively reported improvement. One patient developed hypoesthesia in the right L3 territory (7%) while three patients developed transient paresthesia in the region innervated by the affected nerve (21%).

One patient (7%) developed a transient postoperative ileus that could be managed nonoperatively.

The mean length of hospital stay was 5 days.

### 3.2. Review of the Literature

Our literature search yielded 1216 results; among those, we selected only the pertinent case series of benign retroperitoneal and/or pelvic PNST reporting at least five patients. Clinical, diagnostic, and surgical data collected from these publications have been summarized in Table 2.

**Table 1:** Summary of our case series. Abbreviations: F, female; M, male; N, neurofibroma; S, schwannoma.

	Age, Sex	Origin, Location	Histology	Size (mm)	Symptoms	Surgical Approach	Surgical Time (min)	Blood Loss; Transfusion	Follow-Up (mos)	Clinical Outcome	Residue; Recurrence	Preop Biopsy
Patient 1	55, M	Right obturator nerve, postero-lateral to psoas	S	40	None	Lateral retroperitoneal	300	850 mL, no	108	Right medial thigh hypoesthesia	No, no	No
Patient 2	27, F	Right femoral nerve, pelvis lateral to psoas	S	60	None	Lateral retroperitoneal	240	400 mL, no	108	Ambulating on P.O. day 1; no new deficits	No; no	No
Patient 3	60, F	Left femoral nerve, pelvis anterior to psoas	S	60	Left groin and thigh pain	Lower midline laparotomy; transperitoneal	180	450 mL, no	36	Ambulating on P.O. day 1; no new deficits	No; no	No
Patient 4	40, F	Right obturator nerve, postero-medial to psoas	N	45	Abdominal and groin pain	Midline laparotomy; transperitoneal	120	200 mL; no	30	Ambulating on P.O. day 1; no new deficits; transient paresthesias on left obturator territory	No; no	No
Patient 5	36, F	Sacral plexus (S1-S2), pelvic presacral	S	130	Left sciatic hypoestesia	Lower midline laparotomy; retroperitoneal	480	3000 mL; yes	30	Ambulating on P.O. day 1; no new deficits; paresthesias on sciatic territory (improving)	Yes; no	Yes (open)
Patient 6	56, F	Left sciatic nerve, pelvis lateral to sacrum	S	50	Left sciatic pain	Pfannestiel incision; transperitoneal	420	500 mL; no	24	Ambulating on P.O. day 1; no new deficits; pain remission; paresthesias on sciatic territory (improving)	Yes; no	Yes (open)
Patient 7	60, F	Left genitofemoral nerve, pelvis medial to psoas	S	20	None	Lower midline laparotomy	NA	NA	24	Ambulating on P.O. day 1; no new deficits	No, no	No
Patient 8	46, F	Right sciatic nerve; pelvis lateral to sacrum	S	20	Severe sciatic pain	Laparoscopic converted to lower midline laparotomy; transperitoneal	NA	150 mL; no	20	Ambulating on P.O. day 1; pain remission; no new deficits	No, no	No
Patient 9	45, M	Sacral plexus; midline sacral promontory	S	75	Lower abdominal pain; abdominal mass	Lower midline laparotomy; transperitoneal	150	200 mL; no	18	Ambulating on P.O. day 1; no new deficits	No; no	Yes (percutaneous)
Patient 10	17, F	Right lumbosacral trunk; lateral to sacrum	S	40	Abdominal pain	Lower midline laparotomy; transperitoneal	360	100 mL; no	18	Ambulating on P.O. day 1; no new deficits	No; no	No
Patient 11	42, F	Left femoral nerve, upper pelvis anterior to psoas	S	35	Left thigh pain	Lower midline laparotomy; transperitoneal	150	400 mL, no	16	Ambulating on P.O. day 1; no new deficits	No; no	No
Patient 12	67, M	Left lumbosacral plexus, medial to psoas	S	60	None	Lower midline laparotomy; transperitoneal	360	100 mL; no	12	Transient ileus, managed non-operatively; no new deficits	No; no	No
Patient 13	28, M	Left femoral nerve, lateral to psoas	S	75	Left tight numbness	Lateral retroperitoneal	360	500 mL; no	12	Ambulating on P.O. day 1; unchanged left tight numbness	No; no	Yes (percutaneous)
Patient 14	72, F	Left femoral plexus branch, medial to psoas	S	60	Left inguinal and thigh pain	Midline laparotomy; transperitoneal	540	300 mL; no	3	Ambulating on P.O. day 1; no new deficits; pain remission	No; no	No
Patient 15	75, M	Left femoral nerve, lateral to psoas	S	100	Left sciatic pain (compression)	Lateral retroperitoneal	220	400 mL; no	1	Ambulating on P.O. day 1; no new deficits; pain remission	No, no	Yes (percutaneous)

**Table 2:** Summary of the case series in the literature with more than five cases of benign retroperitoneal PNST reported. All values are means/medians where not specified. Abbreviations: Mos, months; N, neurofibroma; S, schwannoma [9,10,11,12,13,14,15,16,17,18].

Source, Expertise	No. of Patients (% Male)	Age	Follow-Up (mos)	Histology	Size	Pre-Operative Biopsy	Surgical Approach	Resection (% of Cases)	Blood Loss	Preoperative Symptoms	Neurological Outcomes	Recurrence/Regrowth% (Time)	Notes
Regan 1977, abdominal surgery [9]	5 (80%)	55	48	“Neurilemmoma” (100%)	13 cm	20%, misleading	Laparotomy	Macroscopic en bloc (40%), partial (40%)	>3000 mL in 40% of cases	Abdominal pain (60%), abdominal mass (40%)	Severe leg pain (20%)	0%	The authors recommend preoperative arteriography to estimate tumor vascularity. Two cases of infection following percutaneous or transrectal puncture of the tumor.
Guz 1989, abdominal surgery [7]	6 (33%)	45	16	S (50%), N (50%)	NR	50%, misleading	Laparotomy	Macroscopic en bloc (100%)	NR	Radiculopathy (33%), Abdominal pain (33%), abdominal mass (17%), urinary symptoms (17%)	NR	0%	The authors recommend complete surgical excision vs. “enucleation” due to the risk of missing malignant cases
Gubbay 1995, abdominal surgery [10]	5 (40%)	38	73	S (100%)	8 cm	60%, 2/3 non diagnostic	Laparotomy	Macroscopic en bloc (100%)	NR	Abdominal discomfort (60%), abdominal mass (20%), obstructed labor (20%)	Transient motor deficit (20%)	0%	In two cases (40%), resection hindered by significant bleeding
Goh 2005, abdominal surgery [5]	7 (86%)	43	17	S (100%)	7 cm	14%, misleading	Laparotomy	Macroscopic en bloc (100%)	NR	Abdominal discomfort (28%)	NR	0%	No tumor was associated with significant nerve trunks
Li 2007, abdominal surgery [11]	38 (46%)	44	63 (6–404)	S (98%), MPNST (2%)	15 cm	2%, correct	Laparotomy	Macroscopic en bloc (73%), Subtotal (13%), limited (11%)	NR	Abdominal discomfort (50%), back pain (6%)	1% quadriceps paralysis	1% recurrence (3 years)	Large series with long follow-up; important data on disease recurrence
Theodosopoulos 2008, abdominal surgery [12]	5 (40%)	56	35 (6–75)	S (100%)	13 cm	None	Laparotomy	Macroscopic en bloc (80%), subtotal (20%)	NR	Abdominal discomfort (60%), abdominal mass (20%), DVT (20%)	Sensory and motor deficit (20%)	0%	Embolization facilitated resection/dissection in one case. In two cases tumor resected from major nerve root with deficit in one case
Dozois 2009, abdominal surgery, neurosurgery, etc. [13]	46 (50%)	44	-	S (61%), N (37%), ganglioneuroma 2%)	NR	Most patients (% NR)	Laparotomy	Macroscopic intralesional or en bloc	NR	Low back or pelvic pain (50%)	NR	NR	Neurosurgeons/microsurgeons involved in some cases but no separate data for benign and malignant tumors
Strauss 2011, abdominal surgery [14]	28 (25%)	47	39	S (100%)	9 cm	68% (correct)	Laparotomy	Macroscopic en bloc (85%), subtotal (15%)	NR	Abdominal discomfort (29%), abdominal mass (25%)	NR	0%, one malignant transformation (3%)	Neurological outcomes not reported. one case of malignant transformation during FU (patient’s features not specified).
Ningshu 2012, urology [15]	6 (50%)	49	26 (12–48)	S (100%)	6 cm	None	Laparoscopic	Macroscopic resection from nerve trunk (66%), enucleation (33%)	100 mL	Abdominal discomfort	Permanent deficit (33%), transient deficit (33%)	0%	Six cases of obturator nerve schwannoma. In two cases, nerve was sacrified. In four cases, tumor was resected from nerve (with transient deficit in two cases)
Arrabal-Polo 2013, urology [16]	5 (0%)	38	70 (36–120)	S (80%), melanocytic schwannoma (20%)	129 cc	None	Lumbotomy (80%), laparoscopic (20%)	Macroscopic en bloc (100%)	NR	Abdominal discomfort (100%)	NR	0%	One case of melanocytic histology
Zhang 2016, urology [17]	10 (NR)	NR	NR	S (100%)	NR	NR	Retroperitoneoscopic	Macroscopic en bloc (100%)	-	NR	NR	NR	Retroperitoneoscopic technique
Ji 2017, abdominal surgery [18]	26 (23%)	48	15	“Neurilemmoma” (100%)	5 cm	NR	Laparoscopic (60%), laparotomy (40%)	Macroscopic en bloc (100%)	500 mL (laparotomy), 100 mL (laparoscopic)	NR	NR	0%	Comparative series of laparoscopic vs. laparotomic cases
Hajiabadi 2020, neurosurgery and abdominal surgery [19]	16 (50%)	46	26 (3–117)	S (75%), N (19%), Ganglioneuroma (6%)	66 cc	25%, correct	Laparotomy	Microsurgical intracapsular complete (88%), partial (12%)	680	Radiculopathy (44%), back pain (12%)	Pain relief (78%), no improvement (11%); transient leg numbness (6%)	0% (12% stable residue)	First interdisciplinary series with consistent neurosurgical expertise. Most patients with involvement of lumbosacral nerve trunks

The number of patients in the selected series ranged from 5 to 45, with percentages of female patients between 20% and 100%. The average follow-up spanned from 16 to 70 months. The mean tumor diameter ranged from 6 cm to 15 cm. Preoperative biopsies were obtained in 0–68% of cases. Lesions were approached via laparotomy in nine series, laparoscopy in one series, retroperitoneoscopic technique in one series, and mixed techniques in two series. The goal of surgery was macroscopic en bloc resection in eleven series, with rates of complete resection ranging from 40% to 100%; microsurgical nerve-sparing techniques were adopted consistently [19] or occasionally [20] in two series only.

Average blood loss (rarely reported) ranged from 100 mL to 680 mL. Rates of permanent postoperative neurological deficits, when reported, ranged from 0% to 33%. Recurrence rates at the last follow-up were very low (0–1%); only one malignant transformation was observed [14].

## 4. Discussion

### 4.1. General Issues

The retroperitoneal space is an uncommon location for PNST; in particular, retroperitoneal schwannomas and neurofibromas represent less than 3–6% of all PNST [2,4] and 1–15% of primary retroperitoneal tumors [3,21,22]. Due to the compliance of the retroperitoneum, they often grow to giant sizes before becoming clinically evident, usually in the 5–6th decade of life [8] (Table 1 and Table 2). Still, diagnosis is incidental in about half of cases [8]. The most common presenting symptoms are abdominal discomfort and/or distension (around 50% of cases) [11], while neurological deficits are rare and occur in probably less than 3% of patients [11,13]. Nonetheless, providing a correct statistical estimate of preoperative symptoms is difficult due to relevant biases in patient selection: patients with neurological complaints have a higher probability of being referred for neurosurgical evaluation [13,19], as also reflected by our experience (Table 1).

**Figure 1:**
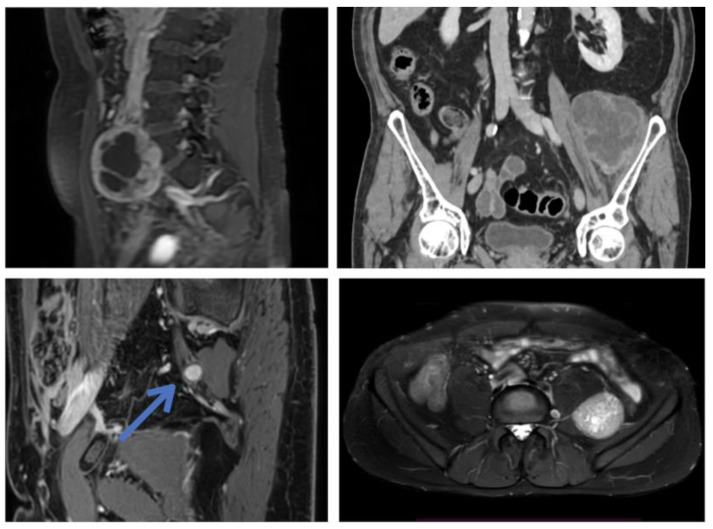
Preoperative MRI images of patients 9 (**top left**), 15 (**top right**), 8 (**bottom left**), and 13 (**bottom right**) showing the heterogeneous radiological features of retroperitoneal PNST.

**Figure 2:**
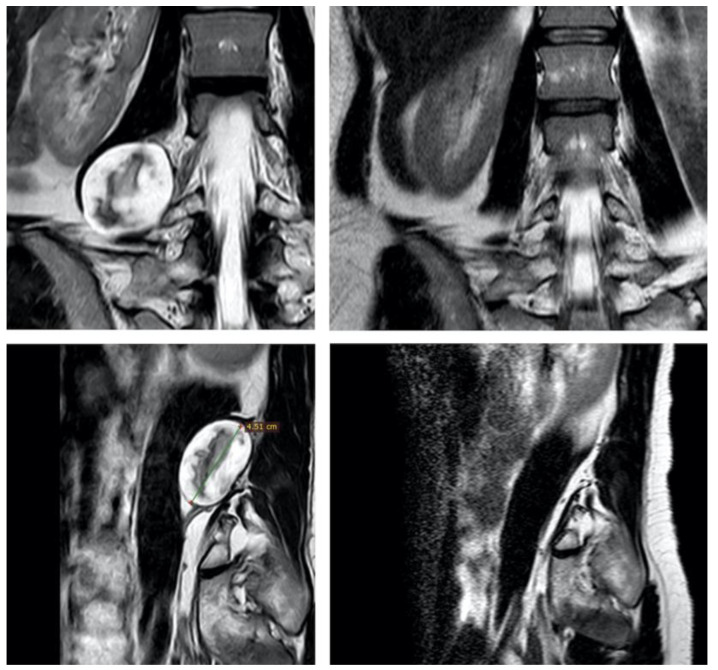
Pre- and post-operative images of patient 4, showing a right obturator nerve neurofibroma.

PNST present some typical radiological features that distinguish them from other retroperitoneal tumors (Figure 1 and Figure 2) [2,14,23,24]. However, it may be more challenging to differentiate them from malignant peripheral nerve sheath tumors (MPNST), which require a completely different treatment [20]. Clinical and instrumental clues can still help in distinguishing between benign and malignant lesions. Red flags are rapid onset of symptoms, progressive motor deficits and/or significant pain at rest [20], a history of NF1, and a radiological appearance characterized by irregular margins, infiltration of nearby structures, heterogeneous signal features, and contrast enhancement [2,23,25,26]. However, the majority of retroperitoneal schwannomas belong to the so-called “ancient” variant, showing signs of cystic degeneration in 66–90% of cases [8,10,11,14,24], and this can give them a heterogeneous MRI appearance, making distinctions less clear [20,25] (Figure 1). In dubious cases, 18F-FDG-PET/CT can be an adjunct in differentiating benign and malignant tumors [23,25,26].

While some authors in the literature have routinely performed preoperative CT or ultrasound guided needle biopsies [13,14], we have reserved that procedure for those cases where diagnostic uncertainties persisted after multidisciplinary preoperative assessment. This is due to multiple considerations: first, especially in cases of giant tumors with areas of degeneration, a needle biopsy can have a suboptimal diagnostic yield and provide inconsistent or misleading responses [7,10,20,27,28]. Moreover, needle biopsies of PNST can be extremely painful for the patient and risk causing nerve damage [20]. Finally, we found that needle penetration through the tumor pseudocapsule generates scar tissue, which can confuse the anatomical plane between tumoral and healthy fascicles, making microsurgical dissection more difficult (patient 13).

We believe that close surveillance can be reasonable for asymptomatic tumors after comprehensive clinico-radiological assessment, reserving surgery in cases of documented growth, which is not uncommon. A large retrospective, multicenter series has estimated an annual tumor volume growth rate of about 10% for retroperitoneal PNSTs, with 23% of patients showing an annual increase in tumor size of more than 20% [8]. It must be kept in mind that surgery for a larger tumor is more challenging and potentially associated with more complications. In our series, indications for surgery in asymptomatic cases were documented growth during follow-up or the patient’s preference.

### 4.2. Surgical Issues

#### 4.2.1. Type of Resection and Complications

**Figure 3:**
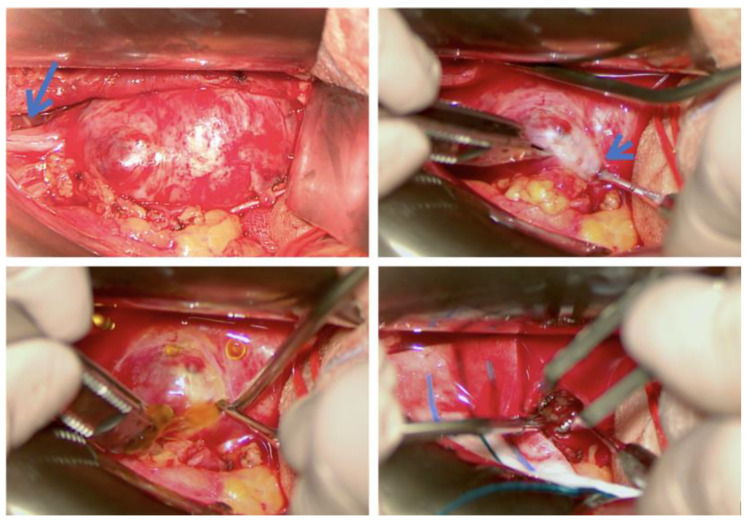
Intraoperative microscopic images demonstrating the technique of extracapsular dissection and piecemeal tumor debulking inside the true capsule (patient 13). (**Top left**): lateral retroperitoneal exposure of the left femoral nerve (arrow) with a 7.5 cm schwannoma; (**top right**): identification of the plane between pseudocapsule and true tumor capsule (arrowhead); (**bottom left**): piercing of the tumor capsule with spillage of cystic fluid; (**bottom right**): piecemeal tumor debulking.

**Figure 4:**
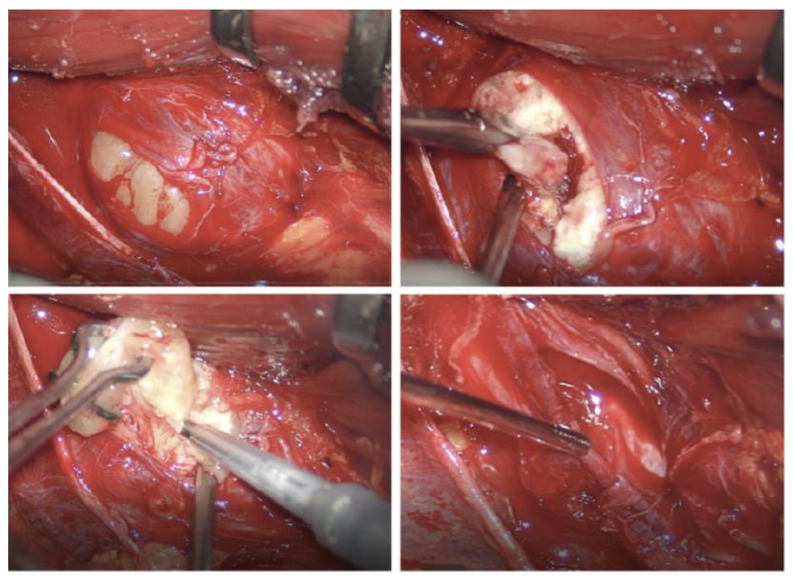
Intraoperative microscopic images demonstrating the technique of extracapsular dissection and piecemeal tumor debulking of a right femoral nerve schwannoma (patient 2). (**Top left**): the pseudocapsule has been incised in a “safe zone” that did not elicit a response when stimulated. (**Top right**, **bottom left**): piecemeal tumor debulking with the aid of an ultrasonic aspirator; (**bottom right**): final product with the tumor completely removed without violating the pseudocapsule.

Most published case series are authored by specialists that deal primarily with abdominal and retroperitoneal pathology, such as general surgeons and urologists (Table 2), and among the articles matching our search criteria, only one was a multidisciplinary series with consistent involvement of micro/neurosurgical expertise [19]. Especially in older publications, the focus has been on radical resection with disease-free margins, citing reasons such as the difficulty of formulating a univocal preoperative diagnosis and the need to obtain R0 tumor removal to prevent recurrence [7,11,12]. Anyway, in a large series (n = 82) with long postoperative patient surveillance (median 63 months) [11], tumor regrowth was observed only once, even if the rate of incomplete resection was 27% (n = 22); no other recurrences or malignant transformations were documented [11]. This questions the need to apply such principles of radicality to the surgical resection of retroperitoneal schwannomas. Moreover, while en bloc resection may be feasible for visceral tumors, it risks causing permanent neurological deficits in retroperitoneal PNST, which are associated with major lumbosacral nerve trunks in more than 20% of cases [8]. Simply trying to preserve the macroscopic continuity of the parent nerve, as advocated by some authors [11,12], does not guarantee an intact postoperative function since the tumor pseudocapsule (which coincides with the interfascicular epinevrium) harbors non-pathologic nerve bundles and nerve-feeding vessels [1,20] that can be damaged when microsurgical techniques are not employed (Figure 3 and Figure 4). For the same reason, en bloc enucleation of PNST from their pseudocapsule is feasible only in cases of small tumors where a cleavage plane between the tumor and healthy fascicles is easily found (an example is case 8 in our series). On the contrary, most larger tumors require internal debulking and progressive piecemeal removal to allow for the identification and development of such a dissection plane.

In the literature, reported rates of permanent neurological complications range from 1% to 33% (Table 2). Unfortunately, however, comparing neurological outcomes of patients operated on with and without microsurgical techniques is difficult, as (1) neurological outcomes are under-reported in general surgical series; (2) most non-microsurgical series do not report the percentage of patients in which the tumor was indeed associated with a significant/major nerve trunk (i.e., patients that are truly at risk of postoperative neurological deficits); (3) some series with involvement of microsurgical expertise mix benign and malignant tumors, despite the obvious differences in treatment goals (nerve-sparing resection vs. radical resection with nerve sacrifice) [13,20]. In such series, rates of postoperative permanent neurological deficits range from 1% (in a large series where the relationship of tumors to nerve trunks was not detailed [11]) to 33% (in a series where all tumors arose from major trunks [15]).

In our series, only one patient (7%) reported a persistent neurological deficit (a numb area on the anterior thigh); in the only other microsurgical series with more than five cases [19], no patients had permanent postoperative neurological deficits (of note, all tumors involved major lumbosacral nerve trunks).

#### 4.2.2. Vascular Features

A consistently reported feature of retroperitoneal benign PNST is its tendency to bleed significantly. Conspicuous intraoperative bleeding is one of the most frequently cited reasons for incomplete resection [29]. In fact, in cases of voluminous PNST, tumor resection and preservation of nerve integrity at the same time are often impossible without piecemeal tumor debulking. This, however, generates continuous oozing, which can make capsular dissection difficult and lead to suboptimal surgical outcomes [20]. Mean blood loss attested to 600 mL in a series of 16 patients [19], but even in the absence of vessel damage, intraoperative blood loss can easily exceed 1 L just from intrinsic tumor bleeding [9,29], as we experienced in one case (Table 1).

For this reason, some authors have occasionally [12] or routinely [29,30,31] employed preoperative embolization when dealing with giant pelvic PNSTs. Aside from a reduction in blood loss [29,30,31], reported benefits include tumor shrinkage with easier capsular dissection [12]. Anyway, since no comparative data are available, it is difficult to estimate the real impact of preoperative embolization on intraoperative bleeding, which remains significant even after embolization in some series [29,30].

Paravertebral retroperitoneal tumors are mainly supplied by lumbar, iliolumbar, and sacral vessels, often forming multiple anastomotic arterial networks [32]. Embolization is associated with a theoretical risk of causing collateral ischemic damage, especially if feeding vessels anastomose with radiculomedullary arteries supplying the spinal cord or if the tumor is supplied by bilateral internal iliac arteries [29].

Balancing the risks and potential advantages outlined above, we decided not to employ preoperative tumor embolization.

A preoperative study with an abdominal angio-CT scan can be useful to assess the relationship of the tumor with the great vessels and to study its vascular features. In some cases, there is an exclusive or predominant supply by a single arterial vessel, which can be recognized and clamped intraoperatively [33] (Figure 1).

### 4.3. Surgical Advances

While most cases in the literature have been approached via laparotomy (Table 2), some authors have adopted a minimally invasive approach based on laparoscopic techniques [18,33,34]. Aside from its better aesthetic results, laparoscopy has been associated with a quicker recovery and shorter hospital stay in a comparative series of retroperitoneal PNST treated either via laparotomy or laparoscopy [18]. Regardless, the 2D vision and the limited maneuverability of multiportal access that characterize laparoscopic surgery are hardly compatible with microsurgical nerve-sparing resection.

**Figure 5:**
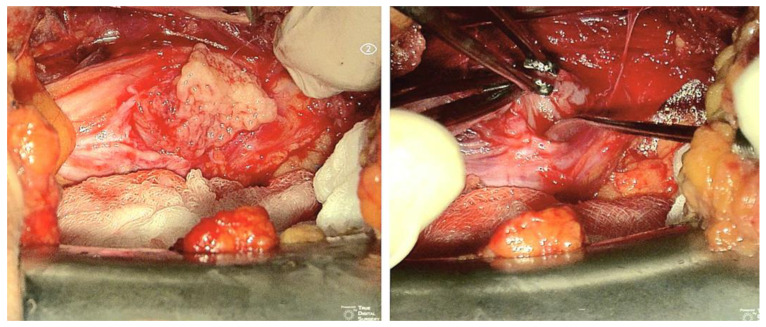
Intraoperative exoscopic images of patient 11 (schwannoma of the left femoral nerve).

Lately, in two of our most recent cases, we introduced in our operative technique the use of a 3D exoscope instead of a traditional operating microscope (Figure 5). The advantages and pitfalls of exoscopic surgery have been diffusely delineated [35]. However, to our best knowledge, this is the first report on the use of such technology in this type of surgery. We appreciated several features that specifically suit the treatment of retroperitoneal PNST, namely: (a) an exoscope is significantly less bulky than traditional operating microscopes and can be moved around and adjusted easily; this is of particular value in abdominal surgeries, which often require ample gestures and a dynamic interplay between operators, assistants, and scrub nurses/technicians; (b) the operator is not constrained to a fixed position: this leads to greater freedom of movement and comfort, especially when the surgeon is not allowed to sit (as happens when dealing with a deep abdominal surgical field); (c) direct visual participation of multiple assistants, as usually required in such kind of surgical approaches, is feasible. In fact, a second and a third assistant can share the same 3D view of the first operator and can help by retracting, aspirating, etc.; on the contrary, with traditional operating microscopes, only the first assistant is able to help accurately. However, our data do not allow us to determine whether the use of the exoscope led to any significant benefit in terms of surgical outcomes.

The limitations of this study include its retrospective, monocentric nature, and the limited number of patients included. On the other hand, our conclusions are somewhat reinforced by the homogeneity in patient management and consistency in surgical technique.

## 5. Conclusions

The surgical management of retroperitoneal PNST is multifaceted and reflects both the complexity of their anatomical location and the risk of neurological damage associated with their resection. Thus, unlike other retroperitoneal tumors, they require a specific microsurgical technique aimed at sparing the functional fascicles of the parent nerve. Multidisciplinary is the key to combining appropriateness in diagnosis and approach choice, safety in resection, and anticipation and management of possible complications.

## Data Availability

All data generated during the present study have been included in the analysis.
